# Trauma and Adversity: Factors Impacting Vulnerability and Resilience Among Older Disaster Survivors

**DOI:** 10.1093/geroni/igaa057.1983

**Published:** 2020-12-16

**Authors:** Molly Davis, Nikki Bellamy

**Affiliations:** 1 George Mason University, Fairfax, Virginia, United States; 2 Center for Mental Health Services (CMHS), Substance Abuse and Mental Health Services Administration (SAMHSA), Rockville, Maryland, United States

## Abstract

Most would agree that older adults represent a highly vulnerable group prior to, during and post disaster. Age-related vulnerabilities often lead into an increased risk for traumatic experiences and post-traumatic stress symptoms after a disaster. Trauma informed principles offer a possible way to reduce the vulnerability of older adults after a disaster. For example, utilizing the trauma informed question “what has happened to you” shifts the focus from a deficit approach and allows for a deeper understanding of the impact of traumatic life experiences on current functioning and reaction to the disaster. It is this understanding of trauma’s impact that may have a role in how older adult disaster survivors view, respond, and recover after a natural disaster (Seery et.al. 2010; Iacoviello & Charney, 2014). In addition, understanding the role of lifetime adversity provides critical insights for disaster planning, reducing vulnerability and promoting resilience among older disaster survivors.

